# The Relative Growth of Invasive *Solanum rostratum* Dunal Decreases with Increasing Competitive Species Richness Regardless of Resource Conditions

**DOI:** 10.3390/plants14233609

**Published:** 2025-11-26

**Authors:** Fa-Zhao Qi, Xiu-Rong Lu, Dong-Pu Wu, Xiao-Jia Zhang, Ying Gao, Lin Geng, Ming-Chao Liu, Yu-Long Feng

**Affiliations:** 1 Liaoning Key Laboratory for Biological Invasions and Global Changes, College of Bioscience and Biotechnology, Shenyang Agricultural University, Shenyang 110866, China; qfz_9708@163.com (F.-Z.Q.); luxr0528@syau.edu.cn (X.-R.L.); 15042845503@163.com (D.-P.W.); zhangxiaojia0512@163.com (X.-J.Z.); 2 Yixian Water Conservancy Affairs Service Center, Jinzhou 121100, China; 18840124411@163.com (Y.G.); 13188121099@163.com (L.G.)

**Keywords:** acquisitive traits, alien species, biotic resistance, field experiment, invasibility, species richness, soil available resources

## Abstract

The biotic resistance hypothesis proposes that species-rich communities are more resistant to biological invasions due to the less available resources for invaders. The variation in available soil resources may affect the invasion resistance of community, but there is little evidence. Using invasive *Solanum rostratum* Dunal, a noxious invader in infertile habitats, and six co-occurring non-invasive species, we conducted a field experiment by testing the relationship between relative growth performance of invasives and richness of resident non-invasives, and the effects of available soil water and nutrients on the relationship. We found that relative aboveground biomass (hereafter relative biomass), relative coverage, community-weighted means (CWM) of specific leaf area (SLA), and photosynthetic rate (*P*_mass_) of *S. rostratum* decreased with increasing resident non-invasive species richness. In contrast, for the non-invasive species, the CWM of SLA and *P*_mass_ significantly increased with increasing resident species richness. However, the available soil water and nutrients exerted limited effects on the relative biomass and coverage of *S. rostratum*. The piecewise structural equation model showed that non-invasive species richness has not only direct negative effects, but also indirect negative effects through promoting non-invasive biomass on the relative biomass of *S. rostratum*. Our findings indicate that the higher resident species richness is fundamental to resist invasion of alien *S. rostratum*, which operates through increasing biomass and resource acquisitive traits of a non-invasive community. Additionally, soil available resources exert limited effects on the invasion resistance. This study suggests the importance of conservation of biodiversity in preventing biological invasions.

## 1. Introduction

Biological invasions are among the most serious threats to global ecosystems, leading to severe degradation of ecological functions and services [1,2]. As one of the most classical theories in invasion biology, Elton’s biotic resistance hypothesis posits that communities with high species richness are more resistant to alien species invasions [3]. Many studies have tested the relationship between invasive alien and native species, but the inconsistent results among them impede our understanding of biotic resistance [4,5,6,7,8,9,10].

Niche complementarity is a main mechanism in biotic resistance that more diverse communities utilize resources efficiently, leaving less available resources for invaders [11]. Thus, the changes of available soil resources may influence the biotic resistance of the native community against invasions. Consistent with this speculation, Cheng et al. (2024) found that biotic resistance of a native community is weakened by experimental drought in their meta-analysis [12]. Inconsistently, in a 4-year experiment, Li et al. (2022) found that nitrogen addition did not affect the relationship between resident species diversity and community invasibility [6]. These contrasting results indicate that the effects of soil resource availability on the biotic resistance may vary with the type and level of resources.

Soil nutrients and water are two main factors influencing invasion of alien plants; however, the effects of their interactions with the community on biotic resistance remain unclear due to a lack of studies [6]. A lot of studies suggested that environments with higher nutrient and water availabilities are more susceptible to invasion by most alien species [13,14,15,16,17]. However, some studies revealed that barren habitats are also invaded by other aliens [18,19,20]. These results indicate that the effects of soil resource availabilities on biotic resistance against invasions may depend on the resources demanding property of the alien species.

*Solanum rostratum* Dunal (Solanaceae) is native to Mexico and the Western United States, and now, has spread to Europe, Australia, Central Asia, and China, becoming an invasive species [21]. It was first discovered in Chaoyang City, Liaoning Province, China, in 1981, and was reported in the Northeast, Inner Mongolia, and Xinjiang of China recently [22]. The seeds of *S. rostratum* can be dispersed by water flow of river, transportation of forage grass, agricultural machinery, animal fur, and wind. The species is commonly found in infertile environments, particularly disturbed grasslands, roadsides, and abandoned land, resulting in serious damage to the natural ecosystems and human activities [22]. To assess the resistance of species richness to the invasion of *S. rostratum*, this study compared the relative growth performance of this species under artificial communities with different non-invasive species richness in an abandoned land. We also regulated rainfall and fertilizer addition within the community and tested the effects of soil resource availabilities on invasion resistance. We hypothesized the following: (1) the relative growth performance of *S. rostratum* would decrease significantly with increasing species richness of non-invasive species in the community; and (2) increasing resource availabilities would enhance the relative growth performance of *S. rostratum* more greatly in species-poor than in species-rich communities.

## 2. Results

### 2.1. Effects of Non-Invasive Species Richness, Water, and Nutrients Treatments on S. rostratum

**Figure 1:**
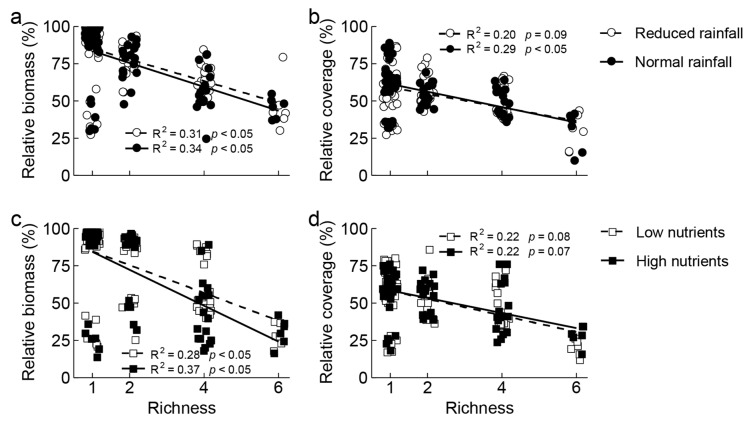
Relationship between relative biomass (**a**,**c**) and coverage (**b**,**d**) of invasive *Solanum rostratum* Dunal (Solanaceae) and non-invasive species richness under different water (reduced and normal rainfall) and nutrients (low and high nutrients per plot) treatments.

The relative aboveground biomass and coverage of invasive *S. rostratum* decreased significantly with increasing non-invasive species richness under all water and nutrient treatments (Figure 1). Compared with the reduced rainfall treatment, normal rainfall treatment decreased relative aboveground biomass of *S. rostratum* in four non-invasive species communities (Figure S3a) and relative coverage of *S. rostratum* in two non-invasive species communities, but increased the relative coverage of *S. rostratum* in one non-invasive species communities (Figure S3b). Compared with the low nutrient treatment, the high nutrient treatment decreased relative aboveground biomass of *S. rostratum* in four non-invasive species communities (Figure S4a) but increased relative coverage of *S. rostratum* in six non-invasive species communities (Figure S4b).

### 2.2. Effects of Non-Invasive Species Richness on CWM Traits and Its Differences Between Non-Invasive and Invasive Species

**Figure 2:**
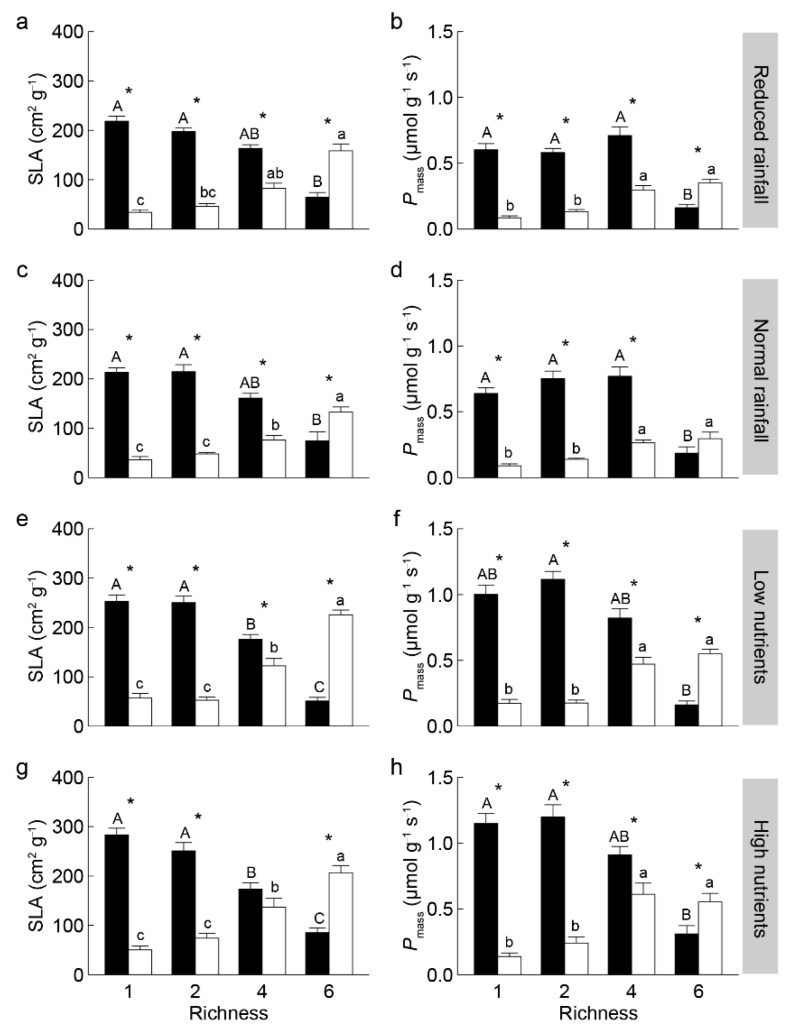
Community-weighted means of specific leaf area SLA (**a**,**c**,**e**,**g**) and mass-based photosynthetic rate *P*_mass_ (**b**,**d**,**f**,**h**) of invasive *Solanum rostratum* Dunal (closed bars) and non-invasive species (open bars) under different water (reduced and normal rainfall) and nutrients (low and high nutrients per plot) treatments. Different uppercase and lowercase letters indicate significant differences among richness levels in invasive and non-invasive species, respectively; * indicates a significant difference between invasive and non-invasive species within the same richness level (*p* < 0.05).

**Figure 3:**
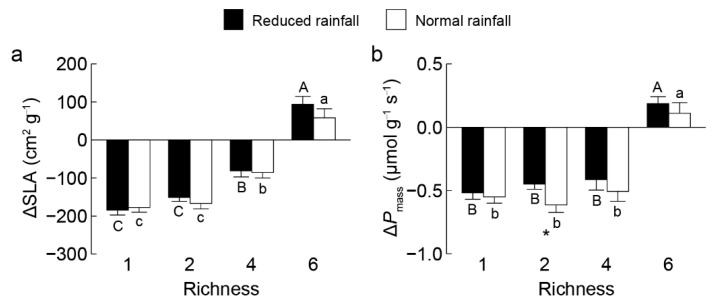
Differences in community-weighted means of specific leaf area ΔSLA (**a**) and mass-based photosynthetic rate Δ*P*_mass_ (**b**) between invasive *Solanum rostratum* Dunal (Solanaceae) and non-invasive species across richness levels under reduced (closed bars) and normal (open bars) rainfall. Uppercase and lowercase letters indicate significant differences among richness levels in reduced and normal rainfall, respectively; * indicates a significant difference between rainfall treatments within the same richness level (*p* < 0.05).

**Figure 4:**
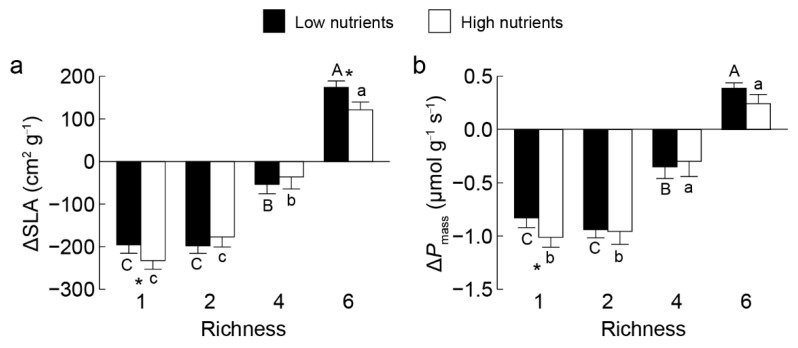
Differences in community-weighted means of specific leaf area ΔSLA (**a**) and mass-based photo-synthetic rate Δ*P*_mass_ (**b**) between invasive *Solanum rostratum* Dunal (Solanaceae) and non-invasive species across richness levels under low (closed bars) and high (open bars) nutrients per plot. Uppercase and lowercase letters indicate significant differences among richness levels in low and high nutrients per plot, respectively; * indicates a significant difference between nutrients treatments within the same richness level (*p* < 0.05).

With increasing non-invasive species richness, the community-weighted means (CWM) of specific leaf area (SLA) and mass-based light-saturated photosynthetic rate (*P*_mass_) significantly decreased for invasive *S. rostratum*, while increased for non-invasive species (Figure 2). The invasive *S. rostratum* had significantly higher CWM traits of SLA and *P*_mass_ in the communities containing one, two, and four (except SLA under high nutrients) non-invasive species; it showed lower values of these traits in the communities containing six non-invasives across all water and nutrients treatments (Figure 2, Figure 3 and Figure 4). We also found that species status (invasive vs. non-invasive) and its interaction with non-invasive species richness significantly affected the CWM traits of SLA and *P*_mass_ (Table S1).

### 2.3. Direct and Indirect Effects of Species Richness and Soil Resources on S. rostratum

**Figure 5:**
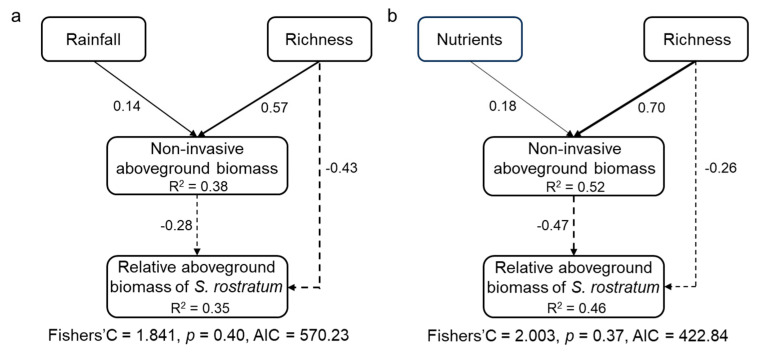
Direct and indirect effects of non-invasive species richness, water (**a**), and nutrients (**b**) treatments on the relative growth of invasive *Solanum rostratum* Dunal (Solanaceae). Only the significant pathways are included in this final model. Solid and dashed lines indicate positive and negative pathways, respectively. Numbers along the lines indicate standardized path coefficients.

The piecewise structural equation model (SEM) results showed that both non-invasive species richness and aboveground biomass had direct negative effects on the relative aboveground biomass of *S. rostratum.* The non-invasive species richness also exerted an indirect negative effect on the *S. rostratum* through promoting the aboveground biomass of non-invasive species. In addition, the water and nutrients availabilities had indirect negative effects on the *S. rostratum* through exerting positive effects on aboveground biomass of non-invasive species, respectively (Figure 5).

## 3. Discussion

Our results showed that relative growth performance (aboveground biomass and coverage) of invasive alien *S. rostratum* significantly decreased with increasing resident species richness, which is consistent with the biotic resistance hypothesis [3]. Consistently, many previous studies revealed negative diversity–invasibility relationships of the community [4,6,8]. However, the inconsistent results were also found in other studies [5,7,23,24]. A possible reason for these conflicted results is that some of these studies did not consider effects of plant abundance on the results. Wang et al. (2022) revealed that the abundance of native species is a main factor influencing invasion resistance of native community [25]. Our study restricted community within the same abundance would provide robust evidence in disentangling the relationship of diversity–invasibility of the community.

The changes in soil resources availabilities may influence niche complementarity among residents in diverse communities [26,27], which may affect the resistance of resident community against invasion. However, our results showed that soil resource availabilities have few effects on the relative growth performance of *S. rostratum* across communities with different species richness. In a meta-analysis, Cheng et al. (2024) found that biodiversity resistance of native community is weakened by experimental drought [12]. Our inconsistent results may be attributed to the lower plasticity of the studied invasive species to soil resources. Invasive species with low-nutrient requirements usually exhibit lower growth plasticity to the soil resources, which is associated with its invasiveness in nutrient-limited habitats [28]. The invasive *S. rostratum* has spread toward the arid or semiarid regions since first found in northeast China, suggesting its low-nutrient requirement [20]. These results suggest that it is essential to consider the nutrient requirements of invasive species when assessing the effects of soil resources on the invasion resistance of non-invasive community.

The increasing evidence showed that the biomass of a native community is a valuable predictor in the invasion resistance [6,20,25]. Although the availabilities of soil resources did not directly affect relative growth performance of *S. rostratum*, it had an indirect effect on the *S. rostratum* through promoting aboveground biomass of non-invasive species. Our piecewise SEM showed that both water and nutrients were positively correlated with aboveground biomass of non-invasives. The increases in aboveground biomass in non-invasives can strengthen their competitive ability against invasive species, thereby enhancing invasion resistance of non-invasive community.

Functional traits, particularly associated with plant growth rates, are often related with the higher environmental resources use efficiency [13,14,29]. Our results showed that with increasing richness of non-invasive species, the CWM of resource-acquisitive traits (SLA and *P*_mass_) significantly increased for non-invasives, but decreased for the invasives. When the number of non-invasive species reached six, the CWM of SLA and *P*_mass_ were significantly higher for the non-invasive than invasive *S. rostratum*, suggesting that persisting the higher species richness (over 6 species per m^2^) would successfully resist invasion of the *S. rostratum*. These results indicate that communities with the higher species richness not only increase niche complementary effect by diverse species, but also enhance the functional resistance by increasing trait superiority of community against invasions. Contrary to our results, recent studies revealed that communities with higher CWM of resource-conservative traits are more resistant to invasion [6,20]. These inconsistent results may be due to the different species included in the communities and different available soil resources among these studies. Although functional traits associated with different growth strategies were found in the invasion resistance among studies, these results suggest that functional traits play a critical role in the invasion resistance of community.

Although a clear negative relationship of diversity–invasibility was found in this study, the experiment with more invasive and non-invasive, particularly native species, is necessary to conduct for testing invasion resistance. Our study involved some non-invasive alien species, which may be different from the native species in the invasion resistance because the aliens may adapt differently to the local habitat as the natives.

## 4. Materials and Methods

### 4.1. Study Sites and Non-Invasive Species

This study was conducted in an abandoned land (41°34′19″ N, 121°9′9″ E; 70 m asl) in Yixian County, Jinzhou, Liaoning Province, China. This region experiences invasion by *S. rostratum* and some other alien species, and featured predominantly sandy soils with relatively low fertility [30]. The study site has a continental monsoon climate, characterized by a mean annual temperature of 8.0 °C and precipitation of 530 mm. The mean growing season (May to August) temperature and precipitation were 22.7 °C and 323 mm, respectively. The experimental land was enclosed to exclude disturbance by large animals. The non-invasive species pool consisted of six species from three families that all co-occurred with *S. rostratum* in field (Table 1) [31]. Seeds of *S. rostratum* and *S. nigrum* were collected in Yixian County, in October 2022, other species were purchased from Shenyang Jinfuyou Seed Co., Ltd. (Shenyang, China).

### 4.2. Experimental Design

In May 2023, the experimental land was thoroughly ploughed and levelled, and all existing vegetation (including rhizomes) was removed. A series of sampling plots (1 m × 1 m) with different non-invasive species richness were established in the experimental land. Plots were 1 m apart from the neighbors. Based on the phylogenetic relationships between the invasive and non-invasive species, we established four gradients of non-invasive species richness: one, two, four, and six non-invasives. Each plot included 21 individuals of non-invasives and 4 individuals of *S. rostratum*. Each *S. rostratum* was surrounded by non-invasive species. The seeds of all species were sown directly into plot. In order to ensure simultaneous germination, the seeds of different species were sown at different time points. The plants in each plot were spaced 14 cm apart from each another. In total, 13 species combinations were established (Figure S1). The other weeds were removed immediately if necessary.

Two gradients of water treatments were implemented after a week of seed germination. The normal water treatment was received natural rainfall and drought treatment was a 50% reduction of the natural rainfall. The reduction of rainfall treatment was achieved by installing semi-circular PVC pipes (12 cm in diameter) above the soil surface with 10 cm to divert half of the rainfall away from the plots. Two nutrients’ treatments were also applied; low and high nutrient treatments were the addition of 300 and 600 g compound fertilizer (available N-P-K was 15–15–15%) per plot, respectively. The fertilizer was dissolved in water first and then sprayed onto the soil surface. We divided the fertilizer addition into six times and added once every 10 days. There were six replicates for each treatment, which included a total of 312 plots (13 plots × 2 water × 2 nutrients × 6 replicates).

### 4.3. Parameter Measurements

In mid-August, light-saturated photosynthetic rate (*P*_max_; μmol m^−2^ s^−1^) was measured using Li-6400 Portable Photosynthesis System (Li-Cor, Lincoln, NE, USA). The light intensity in the leaf chamber was set to 1500 μmol m^−2^ s^−1^, the CO_2_ concentration in the reference chamber was maintained at 380 μmol mol^−1^, and the leaf temperature was set to 25 °C. Specific leaf area (SLA; cm^2^ g^−1^) was determined using mature functional leaves as leaf surface area per gram of dry mass. The mass-based light-saturated photosynthetic rate (*P*_mass_ = *P*_max_ × SLA/10000; μmol g^−1^ s^−1^) was calculated. The coverage (%) of each species was visually measured. Aboveground parts of each species were harvested, and oven-dried at 60 °C for 72 h for measuring aboveground biomass. The relative coverage and aboveground biomass of each species were also calculated.

The CWM of each trait for each species was computed based on the relative coverage [32], and the differences in CWMs between non-invasive and invasive species were also calculated as ΔTrait = CWM of non-invasive trait—CWM of invasive trait.

### 4.4. Statistical Analyses

The linear mixed-effect models (LMM) were fitted with the R package *nlme* to evaluate the effects of non-invasive species richness on the relative aboveground biomass and coverage of invasive *S. rostratum* under different water and nutrients treatments. The plot was treated as a random factor [33]. The LMM were also applied to assess the effects of non-invasive species richness, species status (invasive vs. non-invasive) and their interactions on the CWM traits under different water and nutrients treatments, and to test the effects of non-invasive species richness, water, nutrients treatments, and their interactions on the differences of ΔTrait. Post-hoc pairwise comparisons for treatment combinations were performed using the R package *emmeans* [34]. The piecewise SEM was employed to test direct and indirect effects of non-invasive species richness, soil resources (rainfall or nutrients), non-invasive aboveground biomass, and ΔTrait on the relative aboveground biomass of *S. rostratum* [35]. The SEMs were constructed using linear mixed-effect models where the plot was treated as a random factor. An a priori model incorporating all potential pathways was developed (Figure S2) and then refined by sequentially removing non-significant pathways to obtain the final model. Model adequacy was assessed using the χ^2^ test and Akaike’s Information Criterion (AIC), which were implemented in R package *piecewiseSEM* [35]. These analyses were conducted in R 4.3.3 (R Development Core Team, Vienna, Austria).

## 5. Conclusions

This study provides clear evidence in supporting the biotic resistance hypothesis that the relative aboveground biomass and coverage of invasive *S. rostratum* decreased with increasing non-invasive species richness. The piecewise structural equation model showed that non-invasive species richness has not only direct negative effects by diverse species, but also indirect negative effects through promoting non-invasive biomass by increasing community-weighted means of resource-acquisitive traits on the relative biomass of *S. rostratum*. Soil available resources exert limited effects on the invasion resistance of a non-invasive community. These findings highlight the critical role of species diversity in preventing biological invasions.

## Figures and Tables

**Table 1:** Species list included in the study.

Species	Family	Life Span	Growth Form	Origin	Native Range
*Solanum rostratum* Dunal	Solanaceae	annual	forb	invasive	Mexico, western United States
*Solanum nigrum* L.	Solanaceae	annual	forb	native	China
*Alkekengi officinarum* Moench	Solanaceae	perennial	forb	alien	Mexico, Central America
*Medicago sativa* L.	Fabaceae	perennial	forb	alien	Mediterranean
*Astragalus laxmannii* Jacq.	Fabaceae	perennial	forb	native	China
*Bromus inermis* Leyss.	Poaceae	perennial	grass	alien	Eurasia
*Leymus chinensis* (Trin. ex Bunge) Tzvelev.	Poaceae	perennial	grass	native	China

## Data Availability

All data generated or analyzed during this study are included in this published article (Supplementary Files) and also available from the corresponding author on reasonable request.

## Supplementary Materials

The following supporting information can be downloaded at: https://www.mdpi.com/article/10.3390/plants14233609/s1, Table S1. Effects of non-invasive species richness (*n* = 4), species status (*n* = 2), and their interactions on community-weighted means of specific leaf area (SLA) and mass-based light-saturated photosynthetic rate (*P*_mass_) under different water and nutrients treatments; Table S2. Effects of non-invasive species richness (*n* = 4), water (*n* = 2) or nutrients (*n* = 2) treatments, and their interactions on the differences in community-weighted means of specific leaf area (ΔSLA) and mass-based light-saturated photosynthetic rate (Δ*P*_mass_) (Δtrait = non-invasive − invasive); Figure S1. Schematic diagram of *Solanum rostratum* Dunal and non-invasive species community. Species codes: 0, *Solanum rostratum* Dunal; 1, *Solanum nigrum* L.; 2, *Alkekengi officinarum* Moench; 3, *Medicago sativa* L.; 4, *Astragalus laxmannii* Jacq.; 5, *Bromus inermis* Leyss.; 6, *Leymus chinensis* (Trin. ex Bunge) Tzvelev; Figure S2. An *a priori* conceptual structural equation model (SEM) depicting pathways by which non-invasive species richness and resources (water or nutrients) treatments may influence the relative aboveground biomass of *Solanum rostratum* Dunal. The plot was treated as a random factor in the SEM. For meanings of ΔSLA and Δ*P*_mass_ abbreviations see Table S2; Figure S3. Relative aboveground biomass and coverage of *Solanum rostratum* Dunal across non-invasive species richness under reduced (open bars) and normal (closed bars) rainfall treatments. Reduced rainfall, 50% reduction of rainfall; normal rainfall, ambient rainfall. * indicates significant differences between reduced and normal rainfall treatment within the same non-invasive species richness (*p* < 0.05, linear mixed-effect models); Figure S4. Relative aboveground biomass and relative coverage of *Solanum rostratum* Dunal across non-invasive species richness under low (open bars) and high (closed bars) nutrients treatments. Low nutrients, addition of 300 g fertilizer per plot; high nutrients, addition of 600 g fertilizer per plot. * indicates significant differences between low and high nutrients treatment within the same non-invasive species richness (*p* < 0.05, linear mixed-effect models).
