# Supplementary material for: The Relative Growth of Invasive Solanum rostratum Dunal Decreases with Increasing Competitive Species Richness Regardless of Resource Conditions
Source: Plants (Basel). 2025 Nov 26;14(23):3609. doi: 10.3390/plants14233609 (PMC12693939; doi:10.3390/plants14233609)
Supplement: Supplementary file 1 [file plants-14-03609-s001.zip › plants-3955789-supplementary.pdf]

Table S1 Effects of non-invasive species richness (n = 4), species status (n = 2), and their interactions on community-weighted means of specific leaf area (SLA) and mass-based light-saturated photosynthetic rate (P_{mass}) under different water and nutrients treatments.

		SLA		P_{mass}	
	df	*F*-values	*p*-values	*F*-values	*p*-values
**Reduce rainfall**					
Richness	3	0.5	0.70	8.2	< **0.001**
Origin	1	410.1	< **0.001**	201.1	< **0.001**
Richness × Origin	3	46.3	< **0.001**	12.3	< **0.001**
**Normal rainfall**					
Richness	3	1.4	0.24	8.8	< **0.001**
Origin	1	379.6	< **0.001**	274.3	< **0.001**
Richness × Origin	3	31.0	< **0.001**	11.8	< **0.001**
**Low nutrients**					
Richness	3	0.4	0.79	3.9	**0.01**
Origin	1	227.0	< **0.001**	216.4	< **0.001**
Richness × Origin	3	47.7	< **0.001**	23.5	< **0.001**
**High nutrients**					
Richness	3	0.6	0.65	3.7	**0.01**
Origin	1	214.7	< **0.001**	210.3	< **0.001**
Richness × Origin	3	39.6	< **0.001**	21.2	< **0.001**

Table S2 Effects of non-invasive species richness (n = 4), water (n = 2) or nutrients (n = 2) treatments, and their interactions on the differences in community-weighted means of specific leaf area (ΔSLA) and mass-based light-saturated photosynthetic rate (ΔP_{mass}) (Δtrait = non-invasive – invasive).

		ΔSLA		ΔP_{mass}	
	df	*F*-values	*p*-values	*F*-values	*p*-values
**Water treatment**					
Richness	3	59.0	< **0.001**	20.4	< **0.001**
Water	1	0.1	0.72	3.1	0.08
Richness × Water	3	0.5	0.71	0.4	0.73
**Nutrients treatment**					
Richness	3	53.1	< **0.001**	29.4	< **0.001**
Nutrients	1	0.5	0.47	1.3	0.29
Richness × Nutrients	3	1.0	0.40	0.5	0.69

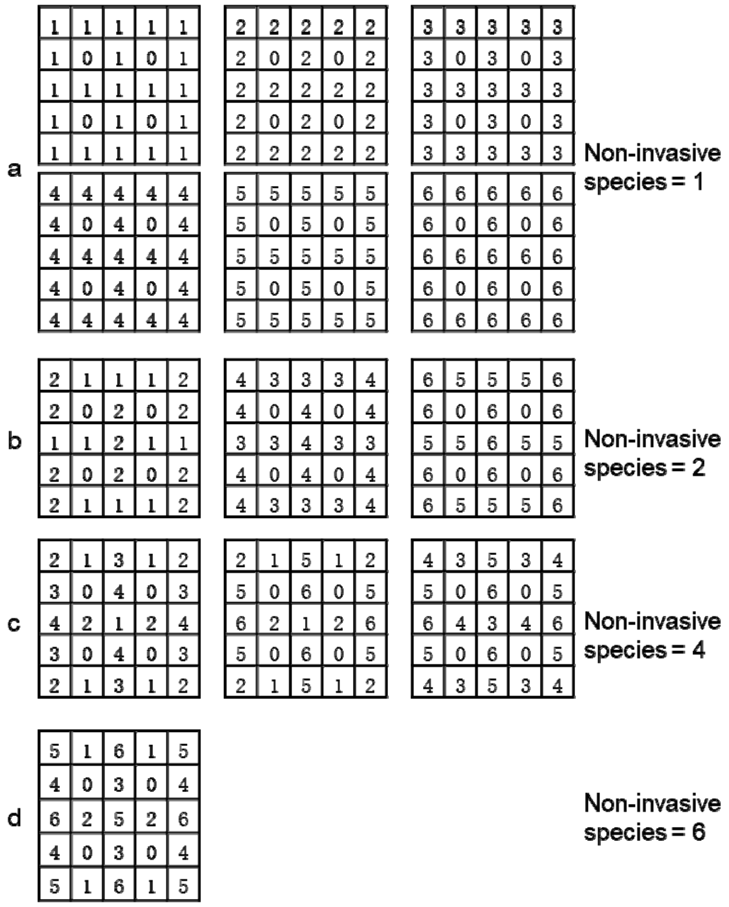

Figure S1 Schematic diagram of *Solanum rostratum* Dunal and non-invasive species community. Species codes: 0, *Solanum rostratum* Dunal; 1, *Solanum nigrum* L.; 2, *Alkekengi officinarum* Moench; 3, *Medicago sativa* L.; 4, *Astragalus laxmannii* Jacq.; 5, *Bromus inermis* Leyss.; 6, *Leymus chinensis* (Trin. ex Bunge) Tzvelev.

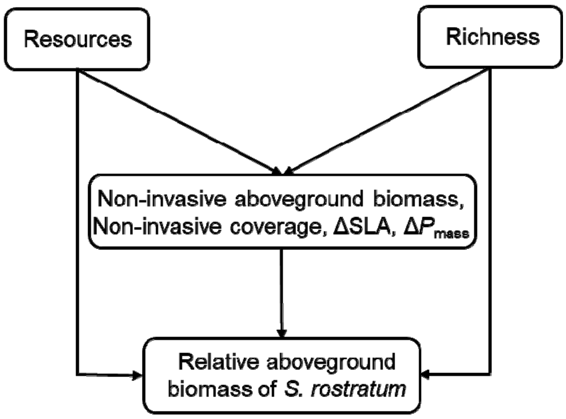

Figure S2 An *a priori* conceptual structural equation model (SEM) depicting pathways by which non-invasive species richness and resources (water or nutrients) treatments may influence the relative aboveground biomass of *Solanum rostratum* Dunal. The plot was treated as a random factor in the SEM. Meanings of ΔSLA and ΔP_{mass} abbreviations see Table S2.

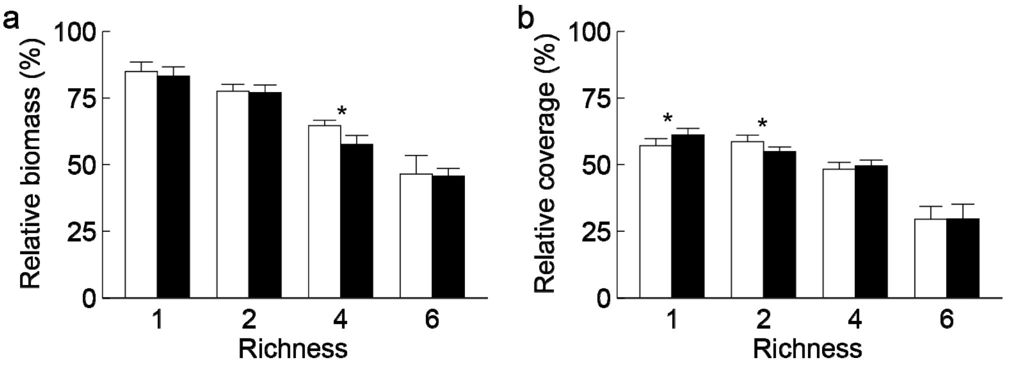

Figure S3 Relative aboveground biomass and coverage of *Solanum rostratum* Dunal across non-invasive species richness under reduced (open bars) and normal (closed bars) rainfall treatments. Reduced rainfall, 50% reduction of rainfall; Normal rainfall, ambient rainfall. * indicates significant differences between reduced and normal rainfall treatment within the same non-invasive species richness ($p < 0.05$, linear mixed-effect models).

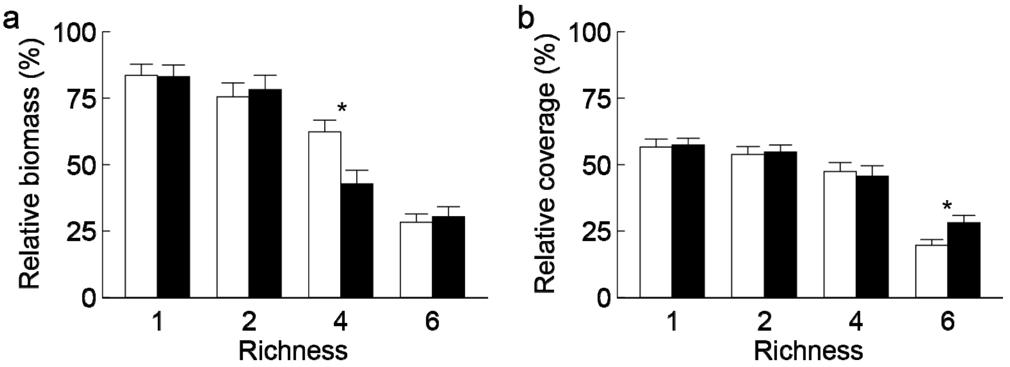

Figure S4 Relative aboveground biomass and relative coverage of *Solanum rostratum* Dunal across non-invasive species richness under low (open bars) and high (closed bars) nutrients treatments. Low nutrients, addition of 300 g fertilizer per plot; High nutrients, addition of 600 g fertilizer per plot. * indicates significant differences between low and high nutrients treatment within the same non-invasive species richness ($p < 0.05$, linear mixed-effect models).
